# pAUL: A Gateway-Based Vector System for Adaptive Expression and Flexible Tagging of Proteins in Arabidopsis

**DOI:** 10.1371/journal.pone.0053787

**Published:** 2013-01-10

**Authors:** Dagmar Lyska, Kerstin Engelmann, Karin Meierhoff, Peter Westhoff

**Affiliations:** Entwicklungs- und Molekularbiologie der Pflanzen, Heinrich-Heine-Universitaet, Duesseldorf, Germany; Purdue University, United States of America

## Abstract

Determination of protein function requires tools that allow its detection and/or purification. As generation of specific antibodies often is laborious and insufficient, protein tagging using epitopes that are recognized by commercially available antibodies and matrices appears more promising. Also, proper spatial and temporal expression of tagged proteins is required to prevent falsification of results. We developed a new series of binary Gateway cloning vectors named pAUL1-20 for C- and N-terminal in-frame fusion of proteins to four different tags: a single (i) HA epitope and (ii) Strep-tag*III*, (iii) both epitopes combined to a double tag, and (iv) a triple tag consisting of the double tag extended by a Protein A tag possessing a 3C protease cleavage site. Expression can be driven by either the 35 S *CaMV* promoter or, for C-terminal fusions, promoters from genes encoding the chloroplast biogenesis factors HCF107, HCF136, or HCF173. Fusions of the four promoters to the GUS gene showed that endogenous promoter sequences are functional and drive expression more moderately and consistently throughout different transgenic lines when compared to the 35 S *CaMV* promoter. By testing complementation of mutations affected in chloroplast biogenesis factors HCF107 and HCF208, we found that the effect of different promoters and tags on protein function strongly depends on the protein itself. Single-step and tandem affinity purification of HCF208 via different tags confirmed the integrity of the cloned tags.

## Introduction

The majority of cellular processes is accomplished and regulated by proteins. To shed light on the precise function of a protein, tools for detection and/or determination of subcellular localization are required. Also, identification and characterization of interaction partners is of great importance as most proteins act in collaboration with other proteins either transiently or in stable complexes. To address all these questions diverse protein tagging strategies have been invented throughout the past years. In-frame translational fusions of the protein of interest and either a reporter protein (e.g. GFP; [1]) or an epitope tag (e.g. hemagglutinin; [2]) are created and introduced into the investigated organism. The Gateway technology (Invitrogen) based on the site-specific recombination mechanism of phage lambda [3] allows rapid cloning of DNA sequences to vectors carrying designated tag sequences. Most of the published Gateway-compatible binary vectors (reviewed by [4]) are designed for constitutive expression of transgenes therefore harboring the 35S promoter of cauliflower mosaic virus (*CaMV*, [5]) or the nopaline synthase (*Nos*) promoter of *Agrobacterium tumefaciens* [6]. This strategy may be disadvantageous for purposes like purification of protein complexes via epitope tagged bait proteins because overexpressed proteins might not be associated with their binding partners. Nevertheless, most purification strategies so far rely on the overexpression of the bait protein in wild-type background with the transgenic protein competing for binding partners with the endogenous protein (e.g. [7]). However, to assure proper function of tagged proteins they should be introduced into respective mutant backgrounds and reconstitute the wild-type phenotype. Furthermore, ubiquitous expression may affect complementation [8], thus demanding for proper regulation of spatial and temporal expression. In this regard, the use of endogenous promoters or promoters of genes with similar expression profiles is more promising.

We designed the binary, Gateway-compatible “pAUL“ vector series for epitope tagging of proteins that are expressed under the control of endogenous *Arabidopsis thaliana* promoters or the 35 S *CaMV* promoter [5]. The primary application is supposed to be detection and purification of nuclear encoded proteins involved in chloroplast-related processes. Thus, vectors with C-terminal tags were generated in the first instance, as N-terminal fusions would be cleaved off toward chloroplast import. The C-terminal tags are combined with *A. thaliana* promoter sequences of genes known to participate in those processes, namely *HCF107* [9], [10], *HCF136* [11], and *HCF173* [12]. To make the vectors applicable for proteins involved in other biological processes vectors harboring the 35 S *CaMV* promoter combined with C-and N-terminal tags were also constructed.

Three epitope tags were utilized for four different C- or N-terminal fusions making possible single-, double- or triple-tagging of proteins of interest. The hemagglutinin (HA) epitope exhibits a small size (27 amino acids for 3x HA) and the availability of effective antibodies make it an ideal tool for detection. Purification can also be carried out in small scales via antibodies or anti-HA matrices and proteins can be eluted competitively by HA peptide or by low pH. The 28-amino acid Strep-tag*III* [13] is an improvement of Strep-tag*II* and has not been described for purification of plant proteins so far. This tag has a strong binding affinity to Strep-Tactin, an engineered streptavidin derivate. Purifications can be performed under flexible binding conditions as Strep-tag*III/*Strep-Tactin interactions are resistant to detergents and varying salt concentrations and do not require the availability of cofactors [13]. Also, the possibility of competitive elution via desthiobiotin makes it a suitable tool for protein and protein complex purification [13]. In the pAUL vector system, the HA epitope and Strep-tag*III* can be used for single-tag-fusions of proteins of interest for detection (HA) or purification (HA and Strep-tag*III*). Double tagging includes both the HA epitope and Strep-tag*III* cloned in series and is supposed to serve for one-step purification via StrepTactin and subsequent detection via the HA epitope. Alternatively, two-step purification via StrepTactin and anti-HA affinity matrix may be carried out if required. Finally, we designed an alternative TAP (tandem affinity purification)-tag. The TAP tag originally developed in yeast consists of two immunglobulin-binding domains of protein A from *Staphylococcus aureus* (ProtA), a tobacco etch virus (TEV) cleavage site and a calmodulin binding site (CBP) [14], but has been modified in the past years (reviewed by [15]). [16] adapted this tag to plant applications and [7] further modified it. We exchanged the CBP by HA for efficient detection of the tagged protein and Strep-tag*III* for purification. The ProtA tag was retained since it displays a strong binding affinity to IgG Sepharose making it well suitable for protein purification. However, the large size of the tag (116 amino acids; ∼13 kDa) may affect the function of the protein fused to it. The TEV cleavage site from the original TAP tag was replaced by the human rhinovirus (HRV) 3C protease cleavage site, which can be processed even at low temperatures according to [7].

Here, we describe cloning of the pAUL vector series. We test the 35 S *CaMV* promoter and promoters from *HCF107*, *HCF136*, and *HCF173* for their activities by quantitative and histochemical GUS assays. Complementation with different promoter/tag combinations is tested using *hcf107.2* [9] and *hcf208* [17] mutants. Corresponding proteins are encoded in the nucleus and transported to chloroplast membranes where they affect thylakoid membrane biogenesis. Whereas HCF107 forms a low abundant high molecular weight complex [10] and is required for expression of the chloroplast-encoded photosystem II subunit PsbH, HCF208 is part of the system IV c-type cytochrome maturation machinery for the *b*_6_ subunit of the cytochrome *b*_6_ *f* complex [18], [19] and fulfills its function as a stable heterodimer transiently interacting with other proteins [20]. Finally, integrity of the different tags is tested by small-scale affinity purification of HCF208 from thylakoid membranes.

## Materials and Methods

### pAUL Vector Construction

Oligonucleotides used for pAUL vector construction are summarized in Table 1.

**Table 1:** Oligonucleotides used for pAUL vector construction.

Oligonucleotide	Sequence (5′to 3′)
pMDC123-H	CCCTCGAGGCGCGCCAAGCTAT
pMDC123-R	GCGCGAGCTCTCGAACCACTTTGTACAAG
T NOS*Pst*I-H	GGCTGCAGCGGAAGATCGTTCAAACATTTG
TNOS*Sac*I/*Hin*dIII-R	CTATGAAGCTTGAGCTCAATTCGATCTAGTAAC
3xHA*Sac*I-H	GACTGGAGCTCTACCCATATGACGTTCCAGAC
3xHAstop*Pst*I-R	GCACTGCAGTTATCAAGCGTAGTCAGGTACGTC
3xHA*Bam*HI-R2	CAGTGGATCCAGCGTAGTCAGGTACGTCG
HA/STREP-1	P-GATCCTGGTCTCATCCTCAATTCGAAAAGGGTGGA
HA/STREP-2	GAACCTCCACCCTTTTCGAATTGAGGATGAGACCAG
HA/STREP-3	P-GGTTCTGGAGGTGGATCAGGTGGTGGATCTTGG
HA/STREP-4	TGAGACCAAGATCCACCACCTGATCCACCTCCA
HA/STREP-5	P-TCTCATCCTCAATTCGAAAAGTGATAAGAGCTCG
HA/STREP-6	AATTCGAGCTCTTATCACTTTTCGAATTGAGGA
HA/STREP-7	CGGGATCCTGGTCTCATCCTCAATTC
HA/STREP-9	CGACTGCAGTTATCACTTTTCGAATTGAGGA
3xHAStrep*IIIXba*I-R	GCTCTAGACTTTTCGAATTGAGGATGAGAC
3cIgG-BD*Xba*I-H	ACTCTAGACTGGAAGTTCTGTTCCAGGGGC
3cIgG-BD*Pst*I-R	CGGGCTGCAGTTATCATACCGAACTCGAATTC
35S*Asc*I-H	GTGAGCTCGGCGCGCCAAGCTTGCATGCCTGCAGGTC
35S*Asc*I-R	GCTCTAGAGGCGCGCCCCTCTCCAAATGAAATGAAC
*HCF107*-Prom-H1	CCGGATTTGGTAGCCACATTCAATGC
*HCF107*-Prom-R1	CCGGCTCGGGGAAGAAGAATGATGG
*HCF107*-Prom-H5-2	GGCGCGCCGGATTTGGTAGCCACATTCAATGC
*HCF107*-Prom-R5	GGCGCGCCGGCTCGGGGAAGAAGAATGATGG
*HCF136*-Prom-H1	CCCTGTTCATTGGAGTCATATCAAGTC
*HCF136*-Prom-R1	CCTCTCTTCTCTTTCTCTCTCCCGC
*HCF173*-Prom-H1	GGCGCGCCTCTCTTACATTTTTGGGCGAACTTG
*HCF173*-Prom-R1	GGCGCGCCAAATGCATACAATTGTTTGTTAAATGAATC
3xHA-*Asc*I/*Pst*I-H	GCACTGCAGGGCGCGCCATGTACCCATATGACGTTCCAGAC
3xHA-*Pst*I-H	GCACTGCAGTACCCATATGACGTTCCAGAC
3xHA-*Asc*I/*Hin*dIII-R	CTATGAAGCTTGGCGCGCCAGCGTAGTCAGGTACGTCG
Strep-*Xba*I-H1	ACTCTAGATGGTCTCATCCTCAATTCGAAAAGGGTGGAGGTTCTG
Strep-*Asc*I-H2	GACGTACCTGACTACGCTGGCGCGCCATGTGGTCT
Strep-*Sac*I/*Asc*I-H-2	GACTGGAGCTCGGCGCGCCATGTGGTCTCATCCTC
Strep-*Pst*I-R	CGGGCTGCAGCTTTTCGAATTGAGGATGAGAC
Strep-*Asc*I-R	CTGGAACAGAACTTCCAGGGCGCGCCCTTTTCG
IgG-*Asc*I/*Bst*XI-H	GACTGCCACCGCGGTGGCGCGCCATGGCCACCATGGCGCAAC
IgG-3C*-Bcu*I-R2	GACACTAGTGGGCCCCTGGAACAGAACTTCCAG

#### Construction of pAUL 1 to pAUL12

Cassette C1 from the pMDC123 Gateway vector [21] was modified prior to cloning of tags and promoters into the plasmid. The att cassette was amplified using primers pMDC123-H and pMDC123-R and then removed from the vector by digestion with *Asc*I and *Sac*I. The PCR product lacking 44 bp stop codon-containing sequence between attR2 site and *Sac*I recognition site was digested using *Asc*I and *Sac*I and cloned into pMDC123 to generate pMDC123-(-)stop. The integrity of the att cassette was tested by sequencing.

The tags were first assembled in pBluescript II (pBSII) KS+ and each fused to the *Nos* terminator, which was obtained form the pC-TAPa plasmid [7] by PCR reaction using primers T NOS*Pst*I-H and T NOS*Sac*I/*Hin*dIII-R. The *Pst*I/*Hin*dIII digested PCR product was ligated into the pBSII phagemid already containing the assembled tags, which were generated as follows.

For vectors containing only the 3x HA tag the DNA sequence from plasmid spa1g3xHA-pBS (provided by Ute Hoecker) [22] was amplified with primers 3xHA*Sac*I-H and 3xHAstop*Pst*I-R and cloned using *Sac*I and *Pst*I generating pBS-3xHA*Pst*I.

The DNA sequence of 3x HA tag cloned into the multiple tags was amplified using primers 3xHA*Sac*I-H and 3xHA*Bam*HI-R2 and cloned into pBSII after digestion with *Sac*I and *Bam*HI generating pBS+3xHA*Bam*HI.

Strep-tag*III* [13] was obtained by annealing of the oligonucleotides HA/STREP-1 to -6 at 90°C for 15 minutes in annealing buffer (0.1 M Tris/HCl, pH 7.5; 1 M NaCl; 10 mM EDTA). The DNA sequence was adapted to plant codon usage. For the generation of 3x HA/Strep-tag*III* the DNA fragment was amplified using primers HA/STREP-7 and HA/STREP-9. The *Bam*HI/*Pst*I digested PCR product was cloned into pBS+3xHA*Bam*HI generating pBS+3xHA/Strep*IIIPst*I.

The 3xHA/Strep*III*/PA tag was created by amplification of the Strep-tag*III* DNA fragment with primers HA/Strep*III*-7 and 3xHAStrep*IIIXba*I-R and amplification of the 2xProteinA tag including 3C protease cleavage site from pC-TAPa using primers 3cIgG-BD*Xba*I-H and 3cIgG-BD*Pst*I-R. Both PCR products digested *Bam*HI/*Xba*I and *Xba*I/*Pst*I respectively were cloned into pBS+3xHA*Bam*HI generating pBS+3xHA/Strep*III*/PA*Pst*I.

The three tag constructs including nos terminator were removed from pBSII by digestion with *Sac*I and ligated into the pMDC123-(-)stop plasmid creating plasmids pMDC123-(-)stop-3xHA, pMDC123-(-)stop-3xHA/Strep*III* and pMDC123-(-)stop-3xHA/Strep*III*/PA. The correct orientation of the tags was verified by restriction analysis.

The 2×35 S *CaMV* promoter and the three endogenous promoters from *HCF107*, *HCF136* and *HCF173* were each cloned by PCR reaction and digestion with *Asc*I into the three pMDC123-(-)stop plasmids containing the tag constructs.

From plasmid pYL436 the 2×35 S *CaMV* promoter was amplified using primers 35S*Asc*I-H and 35S*Asc*I-R. Predicted promoter sequences of genes *HCF107*, *HCF136* and *HCF173* were amplified from genomic DNA of *A. thaliana* Columbia-0 ecotype. The *HCF107* promoter was amplified by adapter PCR using primers *HCF107*-Prom-H1 and *HCF107*-Prom-R1 in the first step and primers *HCF107*-Prom-H5-2 and *HCF107*-Prom-R5 in the second step. Promoters of *HCF136* and *HCF173* were amplified with primers *HCF136*-Prom-H1/*HCF136*-Prom-R1 and *HCF173*-Prom-H1/*HCF173*-Prom-R1 respectively. *Asc*I digested PCR products were ligated to pMDC132-(-)stop-3xHA, pMDC132-(-)stop-3xHA/Strep*III* and pMDC123-(-)stop-3xHA/Strep*III*/PA generating vectors pAUL1 to pAUL12. Correct orientation was checked by restriction analyses. After completion of the vectors promoters and tags were checked by sequencing.

#### Construction of pAUL13 to pAUL16

For the creation of pAUL13 to pAUL16 the modified att cassette from pMDC123-stop was transferred to the Gateway vector pMDC99 [21] generating pMDC99-(-)stop. The Strep-tag*III* sequence was amplified with primers HA/Strep-9 and Strep*III*/*NotSac*-H from vector pBS+3xHA/Strep*IIIPst*I. After digestion with *Not*I and *Pst*I the fragment was ligated to a pBluescript II vector already containing the *Nos* terminator sequence (cloned as described above). Correct fragments were removed from pBSII via a *Sac*I restriction site and ligated to pMDC99-(-)stop creating pMDC99-Strep. Promoters 2×35 S *CaMV*, *HCF107*, *HCF136*, and *HCF173* were extracted from the above-described pAUL vectors via *Asc*I and fused to pMDC99-Strep generating pAUL13 to pAUL16. Correct orientation was checked by restriction analyses. After completion of the vectors promoters and tags were checked by sequencing.

#### Construction of pAUL 17 to pAUL20

N-terminal versions of the pAUL vector series were produced using pMDC32 [21] containing a 2×35 S *CaMV* promoter and nos terminator as a backbone.

3xHA and Strep-tag*III* sequences were amplified from the pAUL2 vector, pN-TAPa [7] served as template for IgG-BD+3C protease cleavage site.

In order to create vectors containing the 3x HA only, the sequence was amplified with primers 3xHA-*Asc*I/*Pst*I-H and 3xHA-*Asc*I/*Hin*dIII-R. For double and triple tag primers 3xHA-*Pst*I-H and 3xHA-*Asc*I/*Hin*dIII-R used. Both PCR products were digested by *Pst*I and *Hin*dIII and ligated to pBSII to create pBS-3xHA-N and pBS-*Pst*I-3xHA-N respectively.

For the single tag Strep-tag*III* was amplified via primers Strep-*Asc*I-H2 and Strep-*Asc*I-R, restricted with *Asc*I and directly ligated to *Asc*I-linearized pMDC32. The Strep-tag*III* sequence needed for the 3xHA/Strep*III* double tag was amplified with primers Strep-*Sac*I/*Asc*I-H-2 and Strep-*Pst*I-R. The *Sac*I/*Pst*I digested fragment was fused to pBS-*Pst*I-3xHA-N resulting in pBS-Strep*III*/3xHA-N. Finally, primers Strep-*Xba*I-H1 and Strep-*Pst*I-R were employed for amplification of Strep-tag*III* needed for the triple tag. The fragment was restricted with *Xba*I and *Pst*I and introduced into pBS-*Pst*I-3xHA-N generating pBS-*Xba*I-Strep*III*/3xHA-N.

The sequence of the 2x Protein A tag and 3C protease cleavage site was amplified with primers IgG-*Asc*I/*Bst*XI-H and IgG-3C-*Bcu*I-R2 and digested with enzymes *Bst*XI and *Bcu*I. The fragment was ligated to pBS-*Xba*I-Strep*III*/3xHA-N digested with *Xba*I and *Hin*dIII (*Bcu*I and *Xba*I form compatible sticky ends) to create pBS-PA/Strep*III*/3xHA-N.

All tag sequences and pMDC32 were restricted with *Asc*I and ligated. After confirmation of the correct orientation the tags were checked by sequencing.

### Cloning of Target Genes to pAUL Vectors

For characterization of the promoters the ß-glucuronidase (*GUS*) gene and the Nos terminator were amplified from the pBI121 vector [23] using primers *GUS*+Term-H and *GUS*+Term-R containing attB sites (Table 2).

**Table 2:** Oligonuleotides used for cloning of target genes.

Oligonucleotide	Sequence (5′to 3′)
*GUS*+Term-H	GGGGACAAGTTTGTACAAAAAAGCAGGCTATGTTACGTCCTGTAGAAACC
*GUS*+Term-R	GGGGACCACTTTGTACAAGAAAGCTGGGTCGATCTAGTAACATAGATGACA
start107 attB1-H	GGGGACAAGTTTGTACAAAAAAGCAGGCTATGCACTTCTTCTTCGTGCCG
107 attB2-R	GGGGACCACTTTGTACAAGAAAGCTGGGTCAGCACCATTTATTCTTCCTC
start208 attB1-H	GGGGACAAGTTTGTACAAAAAAGCAGGCTATGAGTATTCAAATTTGTAATTTC
208 attB2-R	GGGGACCACTTTGTACAAGAAAGCTGGGTCACCTCTGAATTTCTCAGCCATAG

For complementation analyses *HCF107* was amplified with primers start107attB1-H and 107attB2-R (Table 2) using pPEX107PA [10] as a template. *HCF208* was amplified from cDNA obtained by reverse transcription of total RNA isolated from *A. thaliana* wild type Columbia-0 using primers start208attB1-H and 208attB2-R (Table 2).

BP clonase reaction (Invitrogen) between the PCR products and pDONR221 were accomplished according to the Gateway manual creating pENTRY221+*GUS*, pENTRY+*HCF107*, and pENTRY+*HCF208*. Aliquots (5 µl) were transformed into *Escherichia coli* strain *DH5α* using heat shock. The recombinants were selected on LB agar plates containing 50 µg/ml kanamycin. After sequence analysis of the recombined DNA sequence LR clonase reactions (Invitrogen) were performed (according to the Gateway manual) to introduce the genes into the respective pAUL vectors. The ß-glucuronidase gene was introduced into pAUL1, pAUL4, pAUL7, and pAUL10 creating GUSpAUL1, GUSpAUL4, GUSpAUL7, and GUSpAUL10. *HCF107* and *HCF208* were recombined into pAUL1, pAUL2, pAUL3, pAUL6, and pAUL9. Resulting vectors were named HCF107pAUL1, HCF107pAUL2, HCF107pAUL3, HCF107pAUL6, HCF107pAUL9, HCF208pAUL1, HCF208pAUL2, HCF208pAUL3, HCF208pAUL6, and HCF208pAUL9.

Because pAUL vectors as well as the pENTRY221 vectors can only be selected on kanamycin containing media 5 µl of each reaction mixture were digested *Hpa*I or *Eam*1105I respectively in order to linearize the pDONR221+*GUS* vector to avoid its transformation. The vectors were transformed into *DH5α* using heat shock and recombinants were selected on LB agar media supplemented with 50 µg/ml kanamycin. After restriction analyses the correct reading frame of each gene and the tags was checked by sequencing.

### Plant Material, Growth Conditions, and Plant Transformation

All constructs were transformed into *Agrobacterium tumefaciens* strain GV3101 and introduced to *A. thaliana* using the floral dip method [24]. GUS constructs were transferred into wild-type Columbia-0 ecotype. pAUL vectors containing *HCF107* or *HCF208* cDNA were introduced into heterozygous *hcf107.2* (Wassilewskija ecotype) [9] and *hcf208* (Columbia-0 ecotype) [17] plants respectively.

For seed production, protein extraction, spectroscopic measurements, and measurement of GUS activity, plants were grown on soil in a growth chamber operating at a 16 h light/8 h darkness period at a photon flux density (PFD) of ∼50–70 µmol s^−1^ m^−2^ and a constant temperature of 21°C. Protein extracts used for affinity purification were isolated from plants grown under short-day conditions (8 h light/16 h darkness).

Homozygous *hcf208* and *hcf107.2* plants were grown on 0.5×Murashige and Skoog (MS) medium [25] containing 2% (w/v) sucrose and 0.3% (w/v) gelrite (Roth, Karlsruhe, Germany). Seedlings were exposed to a 16 h light/8 h darkness period at a PFD of ∼50–70 µmol s^−1^ m^−2^. Selection of mutant plants exhibiting high chlorophyll fluorescence phenotype was performed in the dark under UV light [11].

Transformants were selected on 0.5×MS medium as described above containing 10 µg ml^−1^ phosphinothricin.

### Measurement of GUS Activity and Histochemical Analyses

GUS analyses were carried out with T1 plants of *A. thaliana* harboring GUSpAUL1, GUSpAUL4, GUSpAUL7, and GUSpAUL10, respectively. Quantitative determination of GUS activity was performed with 15- and 30-day-old plants harvested at midday after 6 hours of illumination according to [26] and [27]. The average values of the data are expressed by medians.

For histochemical analyses either intact 5-day-old seedlings or sections of 3-week-old plants cut manually with a razorblade were transferred into incubation buffer (100 mM Na_2_HPO_4_, pH 7.5; 10 mM EDTA; 50 mM K_4_ [Fe(CN)_6_]; 50 mM K_3_[Fe(CN)_6_]; 0.1% (v/v) Triton X-100; 2 mM 5-bromo-4-chloro-3-indolyl-β-D-glucuronide acid) and vacuum-infiltrated. Samples were incubated at 37°C until they stained blue and fixed in 75% ethanol and 25% acetic acid for 10 minutes. Subsequently, chlorophyll was removed by treatment with 70% ethanol.

### Fluorescence Measurements

For fluorescence measurements complemented *hcf107.2* or *hcf208* plants carrying the respective wild-type gene in pAUL1, -2, -3, -6, or -9 vectors were employed. Chlorophyll fluorescence was imaged with a closed FluorCam FC 800-C controlled by FluorCam 6 software (Photon Systems Instruments) on 3-week-old plants. Experiments were carried out using pre-designed quenching protocol provided by the software.

### Protein Extraction and Western Blot Analysis

Proteins were extracted from the same plants that were also used for fluorescence measurements described above according to [28]. 3 plants from each line were pooled, pestled in liquid nitrogen and immediately transferred to extraction buffer (10 mM Tris/HCl, pH 7.8; 4 M urea; 5% (w/v) SDS; 15% (w/v) glycerol; 10 mM β-mercaptoethanol). The samples were boiled for 4 minutes and cleared by centrifugation at 15,000 g for 5 minutes. Protein concentration was determined using RC DC Protein Assay (Bio-Rad). 50 µg total protein was separated on a 10% SDS-PAGE gel according to [29], transferred to nitrocellulose membranes, and immunoblots were decorated with anti-HCF107 and Anti-HA-Peroxidase (Roche Applied Science).

### Affinity Purification of Fusion Proteins

Leaves of 4- to 6-week-old complemented *hcf208* carrying either HCF208pAUL1, HCF208pAUL2 or HCF208pAUL3 constructs were homogenized in lysis buffer (10 mM Hepes/KOH, pH 7.8; 10 mM MgCl_2_; and 25 mM KCl). Cell debris was separated by Miracloth filtration. The suspension was centrifuged at 4°C until a speed of 5,900 g was reached and then stopped. Pelleted membranes were resuspended in Tris-buffered lysis buffer and solubilized with 1% (w/v) n-dodecyl-ß-D-maltoside (Calbiochem) for 30 minutes at 4°C and a chlorophyll concentration of 1 mg ml^−1^. Unsolubilized material was removed by centrifugation (20 minutes at 4°C and 15,000 g). For each purification aliquots of 200 µl supernatant were added to matrices pre-equilibrated with washing buffer (20 mM Tris/HCl, pH 7.8; 150 mM NaCl; 1 mM EDTA; 0.05% (w/v) n-dodecyl-ß-D-maltoside).

Samples from HCF208pAUL1 (containing the 3x HA tag only) plants were incubated with a bed volume of 50 µl Anti-HA affinity matrix (Roche Applied Sciences) for 1 h at 4°C on a rotator. The matrix was washed with 20 volumes of washing buffer. Proteins were eluted by incubating the affinity matrix three times with 1 volume of elution buffer (1 mg/ml HA peptide (Roche Applied Sciences)) in washing buffer for 15 minutes at 37°C.

HCF208pAUL2 (Strep-tag*III* and 3x HA tag) samples were rotated with 100 µl Strep-Tactin Macroprep (IBA) at 4°C for 1 h, washed with 5 times with 1 volume of washing buffer and eluted with 3 volumes of elution buffer (2.5 mM desthiobiotin (IBA) in washing buffer). Subsequently, the eluate was purified via Anti-HA affinity matrix as described above.

For purification of HCF208pAUL3 (2x ProtA, Strep-tag*III*, and 3x HA tag) samples, respective supernatants were incubated with 50 µl IgG Sepharose (GE Healthcare) for 1 h at 4°C on a rotator. After washing with 20 volumes of washing buffer the matrix was equilibrated with 5 volumes of cleavage buffer (20 mM Tris/HCl, pH 7.8; 150 mM NaCl; 1 mM EDTA; 0.05% (w/v) n-dodecyl-ß-D-maltoside; 1 mM DTT). Cleavage was performed by incubation with 20 units of PreScission Protease (GE Healthcare) in cleavage buffer for 16 h at 4°C on a rotator. The following purification with Strep-Tactin Macroprep was performed according to the procedure described above.

All final eluates were precipitated with 15% (w/v) trichloroacetic acid and separated on 12.5% SDS-PAGE gels according to [29]. Proteins were transferred to nitrocellulose membranes and immunodecorated with Anti-HA-Peroxidase (Roche Applied Sciences) and anti-ATP Synthase.

## Results and Discussion

### Design and Cloning of the pAUL Vector Series

**Figure 1:**
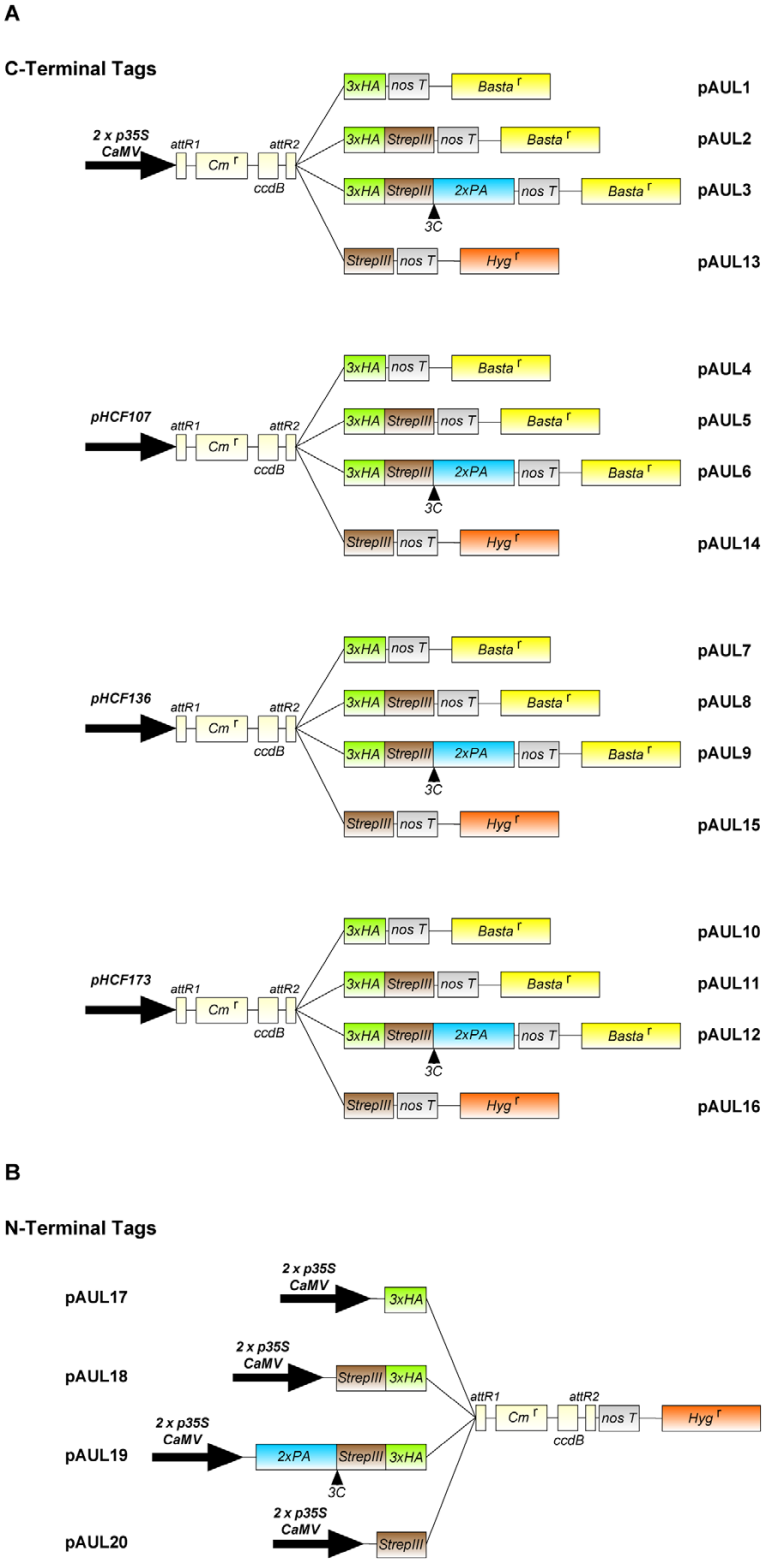
Schematic illustration of the Gateway compatible pAUL destination vector series, showing expression cassettes. (A) C-terminal fusion vectors pAUL1-16. Expression is driven by either 2x p*35* *S CaMV* or endogenous promoter sequences from *A. thaliana* (p*HCF107*, p*HCF136*, p*HCF173*): pAUL1-3 and pAUL13 carry *p35S CaMV*; pAUL4-6 and pAUL14 carry p*HCF107*; pAUL7-9 and pAUL15 carry p*HCF136*; pAUL10-12 and pAUL16 carry p*HCF173*. Protein tags are: 3xHA single tag (pAUL1, 4, 7, 10); Strep-tag*III* single tag (pAUL13-16); 3xHA/Strep-tag*III* double tag (pAUL2, 5, 8, 11); and 3xHA/Strep-tag*III*/ProtA triple tag +3C protease cleavage site (pAUL3, 6, 9, 12). (B) N-terminal fusion vectors pAUL17-20. Vectors carry coding sequences for 3xHA single tag (pAUL17); 3xHA/Strep-tag*III* double tag (pAUL18); 3xHA/Strep-tag*III*/ProtA triple tag +3C protease cleavage site (pAUL19); and Strep-tag*III* single tag (pAUL20).

We constructed a series of 20 binary Gateway-compatible vectors containing different combinations of promoters and tags, named pAUL1-20 (Figure 1). Four different single, double or triple tags were cloned into various backbone Gateway vectors from the pMDC series [21] allowing either C- or N-terminal protein fusions to facilitate protein detection and purification. N-terminally tagged proteins can be expressed under the control of two copies of the 35 S *CaMV* promoter, whereas vectors for C-terminal fusions contain either the two copies of the 35 S *CaMV* promoter for ubiquitous and constitutive expression or one of three endogenous promoters from *A. thaliana*. The endogenous promoters were selected according to the function of the respective genes in chloroplast biogenesis and are described in the following section.

Single tags are the triple HA epitope and Strep-tag*III*. Both tags were combined in order to create the double tag. Addition of a 3C protease cleavage site and the ProtA tag made the triple tag. N- and C-terminal double and triple tags exhibit reverse orientations.

Sequences for C-terminal tags and a *Nos* terminator as well as the four promoters were cloned to the Gateway vector pMDC123 [21] resulting in vectors pAUL1 to pAUL12 (Figure 1A). Plasmid pMDC123 was used as the recipient for the tagging constructs, because this vector does not contain any preexisting promoter nor tag sequences around the Gateway att cassette but unique restriction sites (*Asc*I upstream of the att cassette, *Sac*I downstream of the att cassette) making it suitable for inserting promoter/tag sequences. Moreover, it harbors a bar sequence encoding for phosphinothricin (Basta) resistance driven by a 35 S *CaMV* pormoter.

The C-terminal Strep-tag*III* and promoter sequences were introduced into pMDC99 [21] which corresponds to pMDC123 except it carries a hygromycin resistance instead the bar gene. Those vectors were named pAUL13 to pAUL16 (Figure1A).

If required, promoters from pAUL1 to pAUL16 can be exchanged easily, as they were cloned after the tag sequences by the rare cutting restriction enzyme *Asc*I.

For vectors pAUL17 to pAUL20 N-terminal tags were inserted into pMDC32 [21], which contains a 35 S *CaMV* promoter, a *Nos* terminator and a hygromycin resistance (Figure 1B). These vectors were not tested in this study as we investigated chloroplast-localized proteins whose N-termini are cleaved off upon import into the chloroplast.

Both, N- and C-terminal tag sequences were inserted into the expression cassette in a way that allows easy cloning of sequences according to the Gateway manual (Invitrogen).

### Characterization of 35S *CaMV*, *HCF107*, *HCF136*, and *HCF173* Promoters

The three different promoter regions from genes *HCF107*, *HCF136*, and *HCF173* were selected according to the respective mRNA profiles from the GENEVESTIGATOR database [30]. Experiments from [31] indicate that *HCF136* and *HCF173* mRNAs accumulate to similar levels but are about 4-fold higher than *HCF107* mRNAs. Furthermore, *HCF107* and *HCF173* mRNAs are regulated diurnally, whereas the *HCF136* mRNA levels are stable throughout the day. The putative promoters were defined as sequences upstream of the transcription initiation site of the respective genes ending in regions of ∼1500 bp or until a UTR of the previous gene is reached. 1525 bp of the sequence upstream of *HCF107* 5′UTR, 1401 bp upstream of *HCF136* 5′UTR, and 721 bp upstream of *HCF173* 5′UTR were cloned to pAUL vectors and are referred to as “*HCF107* promoter” (p*HCF107*), “*HCF136* promoter“ (p*HCF136*), and “*HCF173* promoter“ (p*HCF173*) in the following.

**Figure 2:**
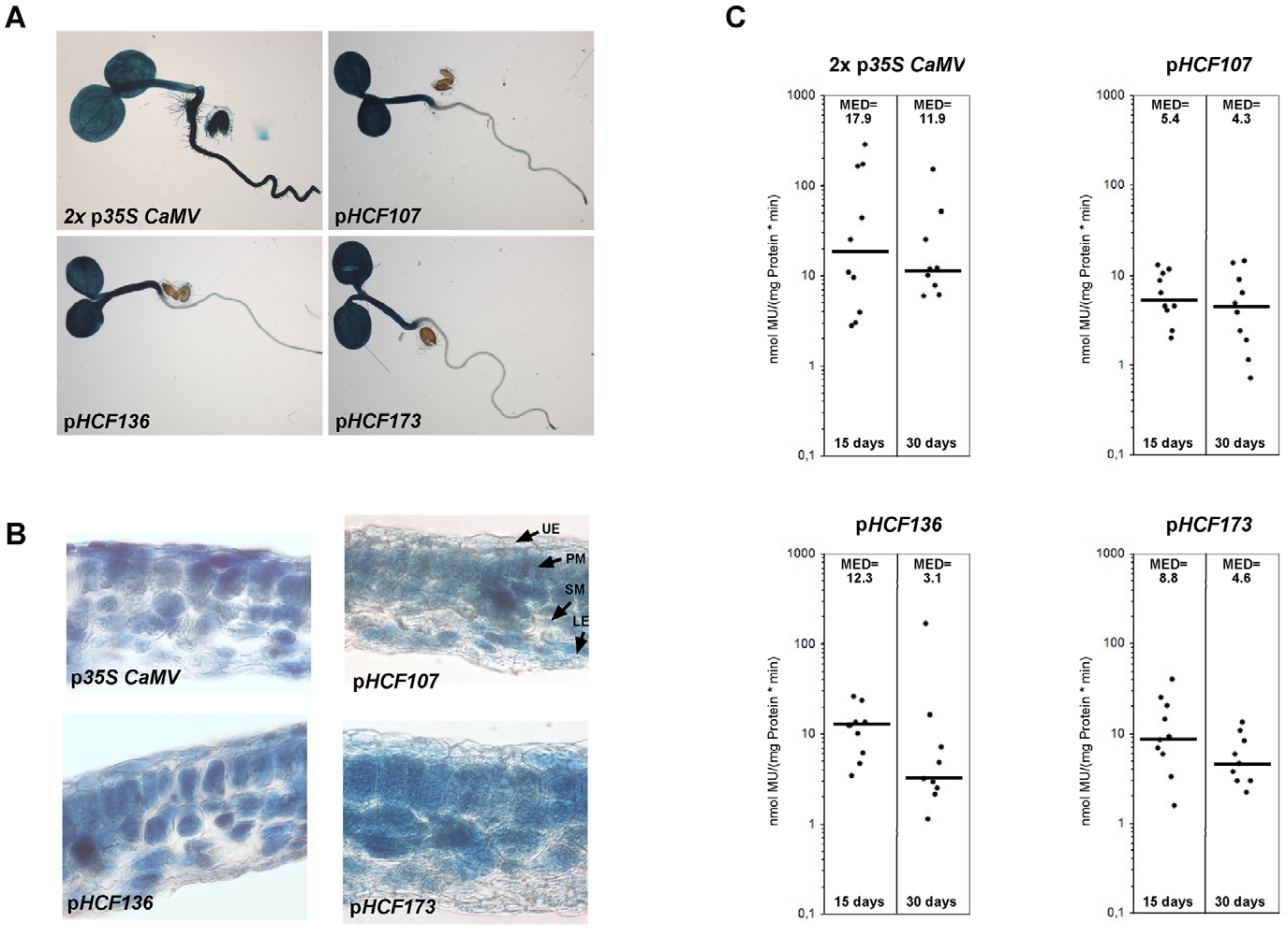
Characterization of promoters 2x p*35S CaMV*, p*HCF107*, p*HCF136*, and p*HCF173* fused to the *GUS* reporter gene. (A) GUS staining of 5-day-old transgenic *A. thaliana* seedlings. (B) Histochemical localization of GUS activity in leaf sections of 3-week-old transgenic *A. thaliana* plants. UE, upper epidermis; PM, palisade mesophyll; SM, spongy mesophyll; LE, lower epidermis. (C) GUS activities in transgenic *A. thaliana* lines. In each case, 10 independent transgenic lines were tested 15 or 30 days after germination. Median values are shown as black bars and indicated at the top of each column. MU, 4-methylumbelliferone.

To test the ability of the selected sequences to serve as promoters and to compare them to the 35 S *CaMV* promoter (p*35S CaMV*) quantitative and histochemical GUS assays were performed. The ß-glucoronidase gene was fused to the four promoter sequences and introduced into wild-type *A. thaliana* plants. As presented in Figure 2 all putative promoter sequences and p*35S CaMV* do function as promoters.

In intact *A. thaliana* seedlings p*35S CaMV* expression is detected in all plant organs, including cotyledons, hypocotyls, roots, and seed coat (Figure 2A). In contrast, p*HCF107*, p*HCF136*, and p*HCF173* are only active in cotyledons and hypocotyls representing the “green” tissue of the seedling but not in roots and seed. This is consistent with the function of HCF107, HCF136, and HCF173 in chloroplast biogenesis [10], [11], [12]. To address tissue specificity of the promoters inside leaves cross sections were prepared (Figure 2B). The 35S *CaMV* promoter is active in all cell types, which agrees with previous studies [32]. However, the staining pattern appears spotted, suggesting that expression is not uniform throughout cell layers and types. In contrast, all endogenous promoters display even staining patterns. Expression of GUS driven by p*HCF107*, p*HCF136*, and p*HCF173* is restricted to palisade and spongy mesophyll cells, which are the chloroplast possessing tissues. Together, these results show that all chosen endogenous promoter sequences are applicable for proper spatial expression of chloroplast-related proteins.

Since another aim of using endogenous promoters was to drive expression more moderately than the 35 S *CaMV* promoter and to ensure proper temporal expression, promoters were also analyzed quantitatively. Data was generated for two different developmental stages (15 and 30 days after germination) from plant material always harvested at the same time of day. In 15 day-old plants, p*HCF107* is the weakest of the endogenous promoters, since p*HCF136* and p*HCF173* exhibit ∼2.3-fold and ∼1.6-fold higher GUS activity than p*HCF107*, respectively (Figure 2C). p*35* *S CaMV* is the strongest promoter, presenting ∼3.3-fold higher activity than p*HCF107* if median values are compared.

However, values for p*35* *S CaMV* are strongly dispersed with their maximum and minimum at 280 and 3 mmol MU/(mg protein*min), respectively, revealing a high variation of expression in individual lines. This characteristic of p*35* *S CaMV* has been reported previously [33]. Furthermore, the presence of multiple 35 S *CaMV* promoter copies is supposed to lead to silencing effects [34], which may occur in vectors that drive expression of the selectable marker by the same promoter and if plants are homozygous for the T-DNA. Expression by endogenous promoters appears more constant throughout different lines suggesting that they do not interfere with any other features of the pAUL vectors.

**Figure 3:**
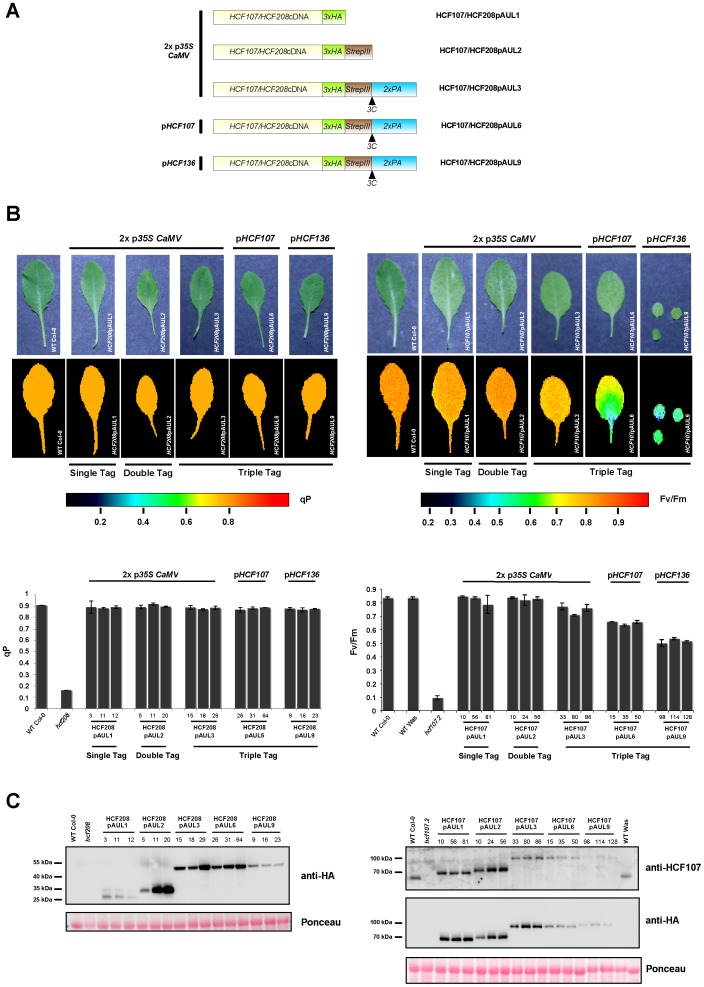
Complementation analysis of *hcf208*and *hcf107.2* with a representative pAUL vector set. (A) Schematic illustration of promoter/cDNA/tag combinations generated for transformation of *hcf107.2* and *hcf208*. (B) Fluorometric analysis of HCF208- and HCF107 complemented plants, wild type and *hcf208/hcf107.2* mutant plants. Pseudo-color images of maximum quantum efficiency of photosystem II (Fv/Fm) are displayed for HCF107 and of photochemical quenching efficiency (qP) are displayed for HCF208. 3 independent transformants were tested for each construct. Values for each line investigated are illustrated in diagrams. (C) Western blot analysis of complemented lines, wild type and mutant plants. 50 µg of crude protein extract were loaded. Membranes were decorated with Anti-HA-Peroxidase antibody for HCF208 and HCF107; HCF107 was also visualized by an HCF107-specific antibody.

30 days after germination promoter activities appear to be decreased compared to values from 15 day-old plants (Figure 3C). p*HCF107* activity is only slightly reduced to ∼80% and therefore is relatively stable. In contrast, p*HCF173* and p*HCF136* activities are drastically reduced to ∼53% and ∼25% respectively, obtaining values similar to p*HCF107*. However, p*35* *S CaMV* expression is relatively stable when individual values are taken into account rather than the median value, which again is not representative due to the high spread. Additionally, p*35* *S CaMV* driven expression is 3-fold higher than the endogenous promoters at that stage.

Altogether, it can be stated that p*HCF107* is suitable for constant and moderate expression of chloroplast-specific proteins, whereas p*HCF136* and p*HCF173* are optimal for proteins involved in early developmental stages (at least up to 15 days after germination) and which are not essential or would even be distracting in later stages. p*HCF136* is adequate for stronger and p*HCF173* for a more moderate early expression. Consequently, before starting cloning of sequences, one should check databases like GENEVESTIGATOR [30] or eFP Browser [35] to select the adequate vector.

### Complementation Analyses of *hcf107.2* and *hcf208*

Tagging of proteins, i.e. attachment of other protein sequences of up to several kDa (e.g. GFP: 27 kDa), and expression driven by foreign promoters may affect the function of fusion proteins. In order to test the influence of the presented tags on protein function and the differential expression by p*35* *S CaMV* or endogenous promoters, we picked two proteins with different functional properties for complementation analyses. Both proteins are encoded by the nucleus and posttranslationally transported into the chloroplast where they perform different functions in chloroplast biogenesis. The HCF107 protein is part of a membrane-associated high molecular weight complex that is involved in stabilization/translation of the *psbH* mRNA [10]. On the other hand, HCF208 is an integral membrane protein that forms heterodimers and small complexes while assisting th *c*-type cytochrome maturation [20].

cDNAs of *A. thaliana* chloroplast biogenesis factors HCF107 and HCF208 were introduced into a subset of pAUL vectors (pAUL1, 2, 3, 6, 9), carrying either a single-, double-, or triple tag and *p35* *S CaMV* or triple tags and p*HCF107* or p*HCF136* (Figure 3A). The constructs were transformed into heterozygous *hcf107.2*/*HCF107* or *hcf208*/*HCF208* background, as homozygous mutants cannot grow photoautotrophically. Transformants were screened for BASTA resistance and homozygous mutant backgrounds on sucrose-supplemented 0.5×MS medium and then were transferred to soil to test their capability to grow photoautotrophically.

The grade of complementation was tested by chlorophyll fluorescence measurements on 3 week-old plants (Figure 3B). Complementation of the photosystem II biogenesis factor *hcf107.2* was indicated by the Fv/Fm ratio, the crucial parameter for photosystem II activity [36] indicating an estimate of the maximum portion of absorbed quanta used in photosystem II reaction centers. Since photosystem II is intact in *hcf208* but the downstream cytochrome *b*_6_ *f* complex is strongly reduced, qP (photochemical quenching) values were determined for indication of complementation. qP displays reduction of variable fluorescence by photosynthetic electron transport processes. From each tested line proteins were isolated and analyzed by Western blot in order to determine levels of fusion proteins.

For HCF208, all promoter/tag combinations are able to fully complement the mutant phenotype (Figure 3B). qP values of both, wild-type and complemented lines are ∼0.9 compared to the drastically lower value 0.16 of *hcf208*. These results indicate that none of the three tags fused to the C-terminus of HCF208 affect its functionality and that all tested promoters are able to drive transgene expression in a way that is sufficient for HCF208 complementation. However, protein levels of transgenic HCF208 vary strongly depending on the construct, as revealed by Western blot analysis using the HA antibody (Figure 3C). Among the transgenes driven by p*35* *S CaMV*, all three independent HCF208pAUL1 lines accumulate very low protein levels unlike HCF208pAUL2- and HCF208pAUL3-constructs. One of the HCF208pAUL2 lines (line 5) also accumulates low amounts of HCF208 compared to the other two lines. There are two possible explanations for low accumulation of HCF208 in pAUL1 lines: either (i) unlike the double- and triple tag, the HA epitope destabilizes HCF208 when attached to its C-terminus or (ii) incidentally all three randomly selected HCF208pAUL1 lines and HCF208pAUL2-5 are silenced. Nevertheless, the residual amounts of HCF208 in these lines are sufficient to complement the mutant phenotype. Also, HCF208 levels in pAUL9 lines correspond to the low levels in HCF208pAUL1 and these lines, too, are fully complemented. As previously tested, the activity of p*HCF136* in pAUL9 decreases during plant development, which accounts very likely for the low protein levels in three week-old HCF208pAUL9 lines. In HCF208pAUL6 lines harboring the transgene driven by p*HCF107*, proteins accumulate to levels similar to HCF208pAUL3. Altogether, it can be stated that protein levels in HCF208pAUL2 (except line 5), -3, and also -6 represent an overexpression of HCF208 exceeding endogenous levels. Unfortunately, no HCF208-specific antibody was available preventing comparison of protein levels in complemented lines to the wild-type situation. In conclusion, these results indicate that (i) the C-terminus of the integral membrane protein HCF208, which forms a large domain extending to the stroma [19] is not prone to attachment of large tags, although it might be influenced by the HA epitope and (ii) expression of HCF208 can be driven by all promoters to complement the mutant phenotype, but the 35 S *CaMV* promoter may be silenced in some lines.

The situation for *hcf107.2* is different in some ways (Figure 3B). Wild-type Fv/Fm values of ∼0.83 compared to ∼0.1 in the mutant *hcf107.2* are only reached by plants carrying HCF107pAUL1 and HCF107pAUL2 vectors, both driving expression by p*35* *S CaMV* and possessing small tags. Extension of the tag sequence by ProtA (pAUL3) decreases Fv/Fm values to ∼0.74. Expression of the triple-tagged protein by p*HCF107* or p*HCF136* (HCF107pAUL6 and HCF107pAUL9) further decreases Fv/Fm values to ∼0.65 and ∼0.53 respectively (Figure 3B). According to these values, HCF107pAUL6 and HCF107pAUL9 plants are paler and smaller than wild type and HCF107pAUL1 to -3 plants. Even three weeks after germination, HCF107pAUL9 plants are very small and hardly produce seed, whereas the defect in HCF107pAUL6 is less severe (Figure 3B).

Western blot analysis was carried out using antibodies against the HA-epitope and HCF107, which was generated in our laboratory. As indicated in Figure 3C, HCF107pAUL1 and HCF107pAUL2 lines over-accumulate the fusion protein compared to wild-type levels, whereas levels of all triple-tagged proteins are significantly lower. Transgenic lines expressing triple-tagged HCF107 under p*35* *S CaMV* control (pAUL3) exhibit about wild-type amounts of HCF107 but, as indicated before, the Fv/Fm ratio displaying photosystem II activity is decreased. This points to an inhibitory effect of the triple tag on protein stability and function. Expression of the triple-tagged protein by pHCF107 and p*HCF136* results in protein levels below wild-type amounts. In HCF107pAUL9 plants HCF107 is hardly detectable.

Former experiments revealed that HCF107 forms a high molecular weight complex [10]. Thus, it is possible that the restriction of protein function by the triple tag may be due to inefficient complex assembly and subsequent degradation of unassembled protein. On the other hand, the protein itself may be unstable independent of its assembly state. In order to achieve nearly wild-type situation, triple tagged HCF107 needs to be overexpressed.

This detailed complementation analysis leads to the conclusion that it strongly depends on the investigated protein which promoter and tag should be chosen for experiments. For HCF208, large tags and expression by endogenous promoters were suitable for complementation, as HCF208 seems to be not required in large amounts for its function and C-terminal tags do not impair protein function, irrespective of their size. In case of HCF107 large tags impair protein function and/or result in destabilization of the protein. Thus, only smaller tags are suitable or overexpression of the incorporated gene is necessary to ensure complementation of the mutant phenotype. This also shows that protein analyses and purification should be carried out in mutant background if possible to show that the protein is not affected by its tag and ectopic expression.

### Purification of HCF208 from Thylakoid Membranes

Integrity of the HA epitope, Strep-tag*III*, and the ProtA tag and purification via these tags were tested on HCF208pAUL1, -pAUL2, and -pAUL3 transgenic lines. The difficulty in purification of HCF208 lies in its feature to be an integral membrane protein. First, crude membranes were treated with 1% n-dodecyl-ß-D-maltoside to solubilize proteins. Subsequently, all purification steps had to be performed in the presence of 0.05% n-dodecyl-ß-D-maltoside to keep proteins soluble.

**Figure 4:**
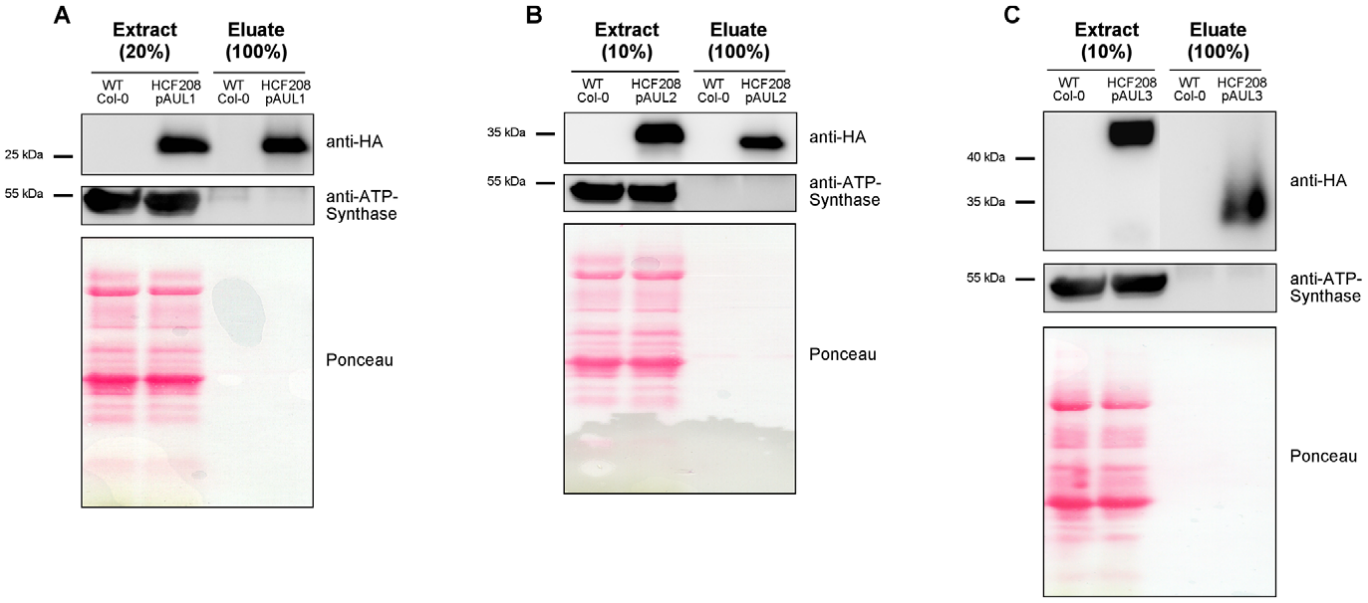
One step and tandem-purification of HCF208. 100 (A) or 200 µg (B, C) chlorophyll aliquots of solubilized membrane proteins were applied for purification. Aliquots of 20 µg chlorophyll from extracts and total amounts of eluates were separated by SDS-PAGE, transferred to a nitrocellulose membrane and immunodecorated with antibodies against the HA tag (Anti-HA-Peroxidase) and ATP-Synthase as a control. (A) One step purification of proteins from wild type and HCF208pAUL1 via the HA epitope and competitive elution. (B) Tandem purification of proteins from wild type and HCF208pAUL2 via Strep-tag*III* and 3xHA (C) Tandem purification of proteins from wild type and HCF208pAUL3 via ProtA tag +3C protease cleavage and Strep-tag*III*.

The western blot analyses presented in the previous chapter indicate that the HA epitope is intact in all tag variants and that it is well suitable for detection using HA antibody. In contrast, detection of the Strep-tag*III* in plant protein extracts at least under our conditions produced an extremely high background. Purification efficiency via anti-HA matrix was tested using solubilized protein extract from HCF208pAUL1. Elution was carried out competitively by the HA-peptide (Figure 4A). The feasibility of the double-, and triple tag for tandem purifications was tested using proteins from HCF208pAUL2 and -pAUL3 transgenic lines. Double-tagged HCF208 protein was loaded on StrepTactin matrix first, eluted competitively by desthiobiotin, and then purified via anti-HA affinity matrix (Figure 4B). Triple-tagged proteins were purified via IgG Sepharose first and eluted by 3C protease cleavage. In the second step, the eluate was incubated with StrepTactin matrix and eluted as described above (Figure 4C). From all purifications significant amounts of HCF208 could be recovered and eluates exhibited no abundant signals (ATP Synthase and Ponceau staining). Successive purification of triple tagged HCF208 from HCF208pAUL3 results in a protein of lower molecular size in the eluate compared to the input, which is consistent with the lack of the ProteinA tag cleaved off by 3C protease treatment (Figure 4C).

These experiments show that both one step and tandem affinity purifications can be carried out with our tagging system always using same buffer conditions and in the presence of detergents. Further, we introduced Strep-tag*III* as a novel epitope that can be used for tagging of plant proteins and purification of proteins and protein complexes due to its property of binding at 4°C and competitive elution via desthiobiotin. Combination with the HA epitope allows easy detection of fusion proteins and, if desired, an additional low-scale purification step. Tandem affinity purification can be carried out using the triple tag via ProtA, which can be cleaved off by 3C protease, and subsequently via Strep-tag*III* with all purification steps at 4°C.

In a recent study, Stoppel et al. used the pAUL11 vector (HCF173 promoter; 3xHA/Strep-tag*III*) for tagging and expressing the chloroplast-localized RNase E (RNE) from *Arabidopsis* in *rne* mutant background. Using the Strep-tag*III* epitope they were not only able to purify the RNE protein, but also to specifically co-precipitate the RNA-binding protein RHON1 [37]. In this way, the pAUL vector system has been proved to be effective tools for the purification of proteins as well as the identification of specific interaction partners.
